# The effect of asafoetida essential oil on myocardial ischemic-reperfusion injury in isolated rat hearts

**Published:** 2018

**Authors:** Hassan Esmaeili, Zahra Hafezimoghadam, Mansour Esmailidehaj, Mohammad Ebrahim Rezvani, Zeynab Hafizibarjin

**Affiliations:** 1 *Department of heart, School of Medicine, Gorgan University of Medical Sciences, Gorgan, Iran*; 2 *Department of Pharmacology, School of Pharmacy, Shahid Sadoughi University of Medical Sciences, Yazd, Iran*; 3 *Department of Physiology, School of Medicine, Shahid Sadoughi University of Medical Sciences, Yazd, Iran*

**Keywords:** Asafetida, Heart, Essential oil, Ischemic-reperfusion injury

## Abstract

**Objective:**

Previous studies reported that asafetida from *Ferula assa-foetida *Linn. species and its essential oil (AEO) have antioxidant effects. In the present study, the effect of AEO was evaluated on ischemic-reperfusion injury in isolated rat hearts.

**Materials and Methods:**

Forty-eight male Wistar rats were divided into 6 groups: 1) control group, 2) vehicle group, 3-5) AEO groups and, 6) carvedilol group. In the control group, hearts were only subjected to 30-min global ischemia followed by 120-min reperfusion. Hearts in other groups were perfused with vehicle (Tween 0.1%), AEO (0.125, 0.25 or 0.50 µL/g heart) or carvedilol (10 µM) for 5 min immediately before the induction of ischemia.

**Results:**

Compared to the control group, myocardial dysfunction was significantly more severe only in group 5 in which a significant increase in left ventricular end diastolic pressure and a significant decrease in left ventricular developed pressure and ± dp/dt. Also, the activities of lactate dehydrogenase and creatine kinase as the markers of myocardial injury were significantly higher only in group 5 compared to control group. The size of infarct and the incidence of irreversible fibrillation did not show any significant differences between the control group and groups 3-5.

**Conclusion:**

These results showed that perfusion of isolated rat hearts with AEO 0.5 µL/g heart, but not at lower concentrations, might worsen myocardial ischemic-reperfusion injury.

## Introduction

Essential oils, naturally-derived aromatic compounds, have gained considerable attention in food industry as food preservatives. These compounds have several biological effects including antioxidant, vasodilatory, anti-inflammatory, anti-allergic, anti-cancer, and antimicrobial activities (Alves-Santos et al., 2016 ▶; Cherkaoui-Tangi rt al., 2016 ▶; Edris, 2007 ▶). Mostly, essential oils are a mixture of generally lipophilic non-volatile and volatile elements with scarcely water-soluble compounds that are synthesized as secondary metabolites in different organs of plants to protect them against external factors such as insects, UV light and pathogens (Donsi and Ferrari, 2016 ▶; Edris 2007 ▶). One of these essential oils is asafoetida essential oil (AEO). 

Asafoetida (Anghoze in Farsi) is an oleo gum resin that is obtained from the excision of the rhizomes and roots of plants named Ferula, especially *Ferula assa-foetida* Linn. (Bagheri et al., 2015 ▶; Mahendra and Bisht, 2012 ▶). *F. assa-foetida* is an herbaceous perennial plant that is native to central Asia, especially Iran and Afghanistan (Bagheri et al., 2015 ▶; Iranshahy and Iranshahi, 2011 ▶). Asafoetida has been traditionally used as antispasmodic, aromatic, carminative, digestive, expectorant, laxative, sedative, nervine, and analgesic agent (Iranshahy and Iranshahi, 2011 ▶; Mahendra and Bisht, 2012 ▶). Experimental studies have also documented that asafoetida has antioxidant (Dehpour et al., 2009 ▶), anti-inflammatory (Iranshahy and Iranshahi, 2011 ▶), antidiabetic (Abu-Zaiton, 2010 ▶; Iranshahi and Alizadeh, 2012 ▶), anticancer (Saleem et al., 2001 ▶), antispasmodic (Bayrami et al., 2013 ▶; Fatehi et al., 2004 ▶; Kiyanmehr et al., 2016 ▶), and hepatoprotective (Dandagi et al., 2008 ▶) effects. It has three major fractions: 1) resin fraction comprising 40 - 64%, 2) gum fraction comprising about 25%, and essential oil fraction about comprising 10 - 17% of asafoetida (Mahendra and Bisht, 2012 ▶). 

Previous studies have shown that asafoetida and its essential oil have antioxidant effects (Saleem et al., 2001 ▶; Kavoosi and Rowshan, 2013 ▶; Kavoosi et al., 2013 ▶; Mallikarjuna et al., 2003 ▶; Safari et al., 2016 ▶). Since excessive generation of free radicals especially at the beginning of reperfusion, plays a critical role in the pathogenesis of ischemic-reperfusion injury (Becker, 2004 ▶; Anaya-Prado et al., 2002 ▶; Bellows et al., 1995 ▶), it might be postulated that AEO could attenuate the severity of ischemic reperfusion injury. Recently, we showed that chronic oral treatment of rats with asafoetida has cardioprotective effect against myocardial ischemic-reperfusion injury at low dose and cardiotoxic effects at high dose (Esmailidehaj et al., 2014 ▶). 

As oral pretreatment of rats with asafoetida oleo gum resin has dual effects on the severity of myocardial ischemic-reperfusion injury (Esmailidehaj et al., 2014 ▶), it was decided to investigate the outcome of perfusion of isolated rat hearts with solution containing AEO on myocardial ischemic-reperfusion injury. In this study, the cardiac dysfunction, size of infarct, incidence percentage of irreversible ventricular fibrillation and some biochemical parameters were evaluated. 

## Materials and Methods

**Chemicals**

Diagnostic kits for lactate dehydrogenase (LDH) and creatine kinase (CK) were obtained from Ziest Chem Diagnostics (Tehran, Iran). 2,3,5-triphenyltetrazolium, sodium pentobarbital, sodium dodecyl sulfate (SDS), thiobarbituric acid, and tetramethoxypropane were obtained from Sigma-Aldrich (St. Louis, Mo, USA). Butylated hydroxytoluene, n-butanol, pyridine, sulfanilamide, orthophosphoric acid, N-naphthylamide hydrochloride, sodium nitrite, and acetic acid were purchased from Merck Company (Germany). All other chemicals were purchased from local companies and were of the highest grade. 

**Preparation of AEO**

Asafoetida was collected from Dorbid area (Yazd, Iran) at the end of spring in 2017. It was identified at the Department of Botany, Faculty of Pharmacy, Shahid Sadoughi University of Medical sciences, Yazd, Iran. A voucher specimen (A2343) was deposited at the herbarium of the Herbal Medicine Research Center of Shahid Sadoughi University of Medical Sciences, Yazd, Iran.

In brief, 100 g asafoetida was powdered and soaked in 500 ml distilled water for 48 hr. Then, its essential oil was extracted and isolated using Clevenger apparatus for 6 hr, yielding 4 mL yellowish essential oil (4% v/w). The water content of essential oil was removed by sodium sulfate. Finally, essential oil was a kept in a dark container at 4^º^C.

**Animals**

To perform this study, male Wistar rats weighing 300-350 g, were used. All animals were housed under standard 12/12 hr light/dark cycle condition in an air-conditioned colony room with free access to standard rat chow and water *ad libitum*. All experimental procedures were done according to the Guide for the Care and Use of Laboratory Animals and approval of the Ethics Committee of Shahid Sadoughi University of Medical Sciences, Yazd, Iran (NO. 3495).

**Experimental Grouping**

Forty-eight male Wistar rats were divided into six groups as follows:

Control Group (Con): In this group, hearts were only perfused with Krebs solution throughout the procedure and subjected to 30 min global ischemia followed by 120 min reperfusion. 

Vehicle group (Veh): In this group, hearts were perfused with 50 mL vehicle (containing Tween 0.1%) immediately before induction of global ischemic-reperfusion injury.

Asafoetida essential oil 0.125 group (E0.125): In this group, hearts were perfused with AEO 0.125 µL/g heart (in 50 mL Krebs solution) immediately before induction of global ischemia ischemic-reperfusion injury.

Asafoetida essential oil 0.25 group (E0.25): In this group, hearts were perfused with AEO 0.25 µL/g heart (in 50 mL Krebs solution) immediately before induction of global ischemic-reperfusion injury.

Asafoetida essential oil 0.50 group (E0.50): In this group, hearts were perfused with AEO 0.50 µL/g heart (in 50 mL Krebs solution) immediately before induction of global ischemic-reperfusion injury.

Carvedilol group as the positive control group (Carv): In this group, hearts were perfused with 50 mL Krebs solution containing carvedilol (10 µM) immediately before induction of global ischemic-reperfusion injury (Yue TL et al., 1998 ▶).

**Isolation and perfusion of the hearts**

Animals were anesthetized by sodium pentobarbital (50 mg/kg, i.p.) and administered with 1000 IU (i.p.) of heparin to prevent coagulation in the coronary vessels during removing of the heart. Then, abdomen and thorax were excised and the heart was removed, weighted, and retrogradely perfused using Langendorff apparatus. Perfusion solution was Krebs-Henseleit buffer containing (mM) NaCl (118), glucose (11), NaHCO_3_ (25), KCl (4.7), MgSO_4_ (1.2), KH_2_PO_4_ (1.2), and CaCl_2_ (1.25). The temperature of the perfusion solution was maintained at 37 °C and gassed with 95 % O_2_ and 5% CO_2_. In order to continuously record the intraventricular pressures using Power lab data acquisition system (ADInstruments, Australia), a water-filled balloon connected to a pressure transducer was inserted in the left ventricle via left atria. Then, the volume of balloon gradually increased to obtain the left ventricular end-diastolic pressure (LVEDP) of around 4-7 mmHg. Two electrodes were placed on the apex and on the base of the hearts to continuously record their electrical activity. 

Following 20 min of stability, the hearts in control group were subjected to 30-min global ischemia followed by 120-min reperfusion. Similar to the control group, the hearts in other groups were perfused with vehicle, essential oil (0.125, 0.25 or 0.50 µL/g heart) and carvedilol (10 µM), 5 min before induction of 30-min global ischemia and 120-minu reperfusion.

**Measurement of cardiac function parameters**

Cardiac function parameters including heart rate, LVEDP, left ventricular developed pressure (LVDP), coronary flow (CF), and the rate of the maximum and minimum pressure changes (±dp/dt) were measured before and during the ischemia and reperfusion times using Power Lab data acquisition system (Lab Chart 7, ADInstrument, Australia).

**Measurement of biochemical parameters in coronary effluent**

In order to measure the activity of lactate dehydrogenase (LDH) and creatine kinase (CK) and the contents of malondialdehyde (MDA) and nitric oxide (NO) in the coronary effluent, one sample was collected at the 5th minute of reperfusion and maintained at -70 °C. The activities of CK and LDH were measured using commercial kits. The content of MDA in the coronary effluent was measured according to the thiobarbituric acid-reactive substances (TBARS) concentration (Jemai et al., 2008 ▶). In brief, 0.1 mL of the sample was added to a falcon tube and mixed with 0.1 mL of SDS 8.1%, 0.75 mL of acetic acid 20%, and 0.8 mL of thiobarbituric acid 0.8%. To prevent any changes in lipid peroxidation, 10 µL butylated hydroxytoluene in ethanol 100%, was added and the volume reached 2 mL by adding distilled water. Next, it was homogenized and kept at 95ºC for 60 min. After cooling, 25 mL n-butanol/pyridine (15:1) and 0.5 mL distillated water were added and vortexed. Finally, TBARS was measured at 532 nm in n-butanol/pyridine phase. Tetramethoxypropane was used as the standard.

The content of nitrite in coronary effluent was measured as marker of NO (Arora, Das, and Srivastava, 2009 ▶). In brief, 50 µL of the sample was mixed with 50 µL of sulfanilamide 1% in orthophosphoric acid 2.5% and 50 µL of N-naphthylamide hydrochloride 0.1% in orthophosphoric acid 2.5% and kept at 37ºC for 30 min. Finally, the absorbance was read at 550 nm. Sodium nitrite (0, 5, 10, 25, 50, 75, and 100 µM) was used as the standard. 

**Determination of infarct size**

At the end of reperfusion, the hearts were freezed at -20°C and then cut into 2-mm sections. To determine the size of infarct, sections were stained with 2,3,5-triphenyltetrazolium chloride and then immersed in 10% formalin to increase the contrast between the infarcted area (white color) and viable area (red color). Afterward, photos were taken from both sides of sections and the average of cross-sectional area of the infarct zone was calculated using Photoshop 8 software. The infarct size was expressed as the percentage of the ventricle whole area. 

**Determination of AEO concentration**

In a pilot study, we decided to choose the concentrations of essential oil that do have significant effects on the intraventricular pressures of isolated rat hearts. It was concluded that AEO at concentrations of less than 0.50 µL/g heart, had no significant effects on intraventricular pressures of isolated hearts under Langendorff apparatus. Higher concentrations led to severe bradycardia or cardiac arrest. Finally, AEO was used at 0.125, 0.25 and 0.50 µL/g heart. AEO was dissolved in 50 ml Krebs solution using Tween 0.1% and then imported into the coronary artery before induction of global ischemic-reperfusion injury.

**Statistical analysis**

Hemodynamic, biochemical and infarct size data were shown as Mean±SEM. The irreversible ventricular fibrillation data were shown as the incidence percentage. The means were analyzed by one-way and two-way ANOVA. The incidence percentages were analyzed by Fisher’s exact test. Graphpad Prism version 6.00 for Windows (Graphpad Software, La Jolla California USA) was used. A p<0.05 was considered statistically significant.

## Results

**Effect of AEO on cardiac dysfunction following ischemic-reperfusion injury**

**Figure 1:**
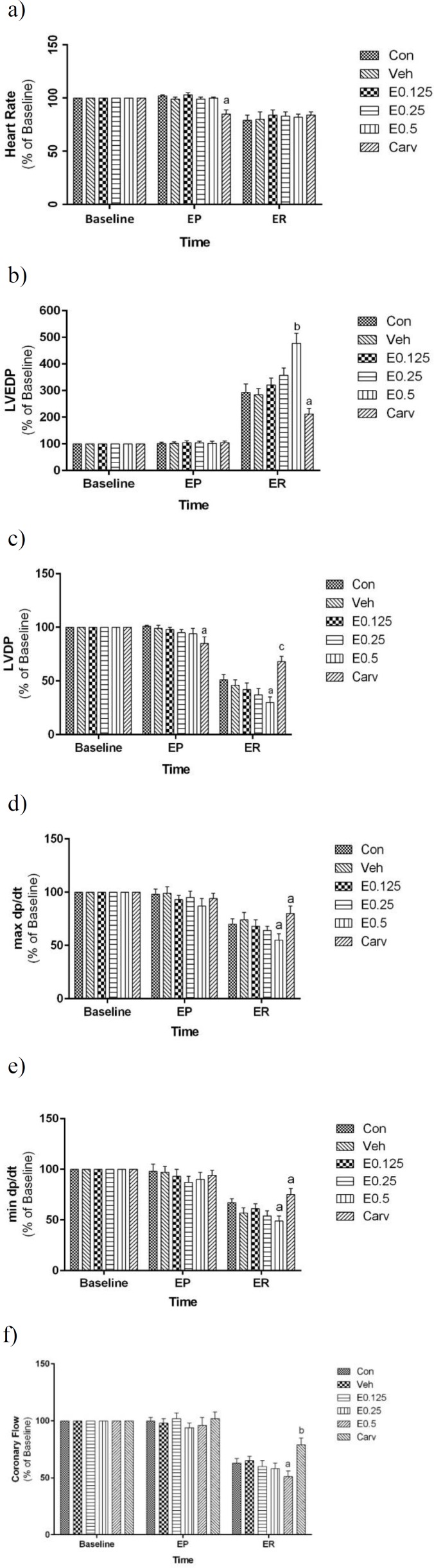
The effect of AEO on cardiac functional parameters and coronary flow following ischemic-reperfusion injury in isolated rat hearts. (a) heart rate; (b) left ventricular-end diastolic pressure (LVEDP); (c) left ventricular developed pressure (LVDP); (d) and (e) maximum and minimum of pressure changes (±dp/dt), respectively; (f) coronary flow. EP, at the end of 5-min perfusion with vehicle, AEO or carvedilol; ER, at the end of 120-min reperfusion; Con: control group; E0.125, E0.250 and E0.5: groups perfused with AEO 0.125, 0.250 and 0.50 µL/g heart in 50 ml Krebs solution immediately before global ischemia, respectively; Carv: carvedilol group (10 µM). Data are shown as mean±SEM. n=8 in each group. ^a^ p<0.05; ^b^ p<0.01 and ^c^ p<0.001 show significant differences as compared to control group

Figure 1 represents the effect of AEO on the cardiac dysfunction following ischemic–reperfusion injury in isolated rat hearts. This Figure represents the percent changes as compared to the base time.

Heart rate was reduced to zero during ischemic time in all experimental groups. Compared to the base time, heart rate was significantly reduced in all groups during reperfusion time (Figure 1a), but there was not any significant difference among the experimental groups at the end of 120-min reperfusion. Heart rate was only significantly decreased in carvedilol group during perfusion time.

LVEDP did not show any significant differences among the experimental groups during baseline and perfusion times (Figure 1b). LVEDP was significantly increased in all groups during ischemic and reperfusion times that was significantly higher only in E0.50 group and lower in carvedilol group compared to the control group (Figure 1b). As mentioned above, carvedilol-treated group was considered the positive control group. 

As Figure 1c depicts, LVDP was reduced to zero during ischemic time in all groups. LVDP was markedly increased during reperfusion in all groups which was significantly lower only in E0.50 group and higher only in carvedilol group compared to the control group. 

Figures 1d and c indicate that ±dp/dt was not significantly different among groups at the end of perfusion time. It was reduced to zero in all groups during ischemic time. ±dp/dt significantly increased in all experimental groups during reperfusion time that was significantly lower only in E0.50 group and higher only in carvedilol group compared to the control group. 

Figure 1f indicates that the coronary flow was not significantly different among different groups during baseline and perfusion times. It was reduced to zero throughout the ischemia in all groups. Although the rate of coronary flow at the end of 120-min reperfusion in all experimental groups, was markedly lower than that of base time, it was significantly lower only in E0.50 group and higher only in carvedilol group compared to the control group. 

**Effect of AEO on coronary flow, infarct size and the incidence of irreversible fibrillation**

**Figure 2:**
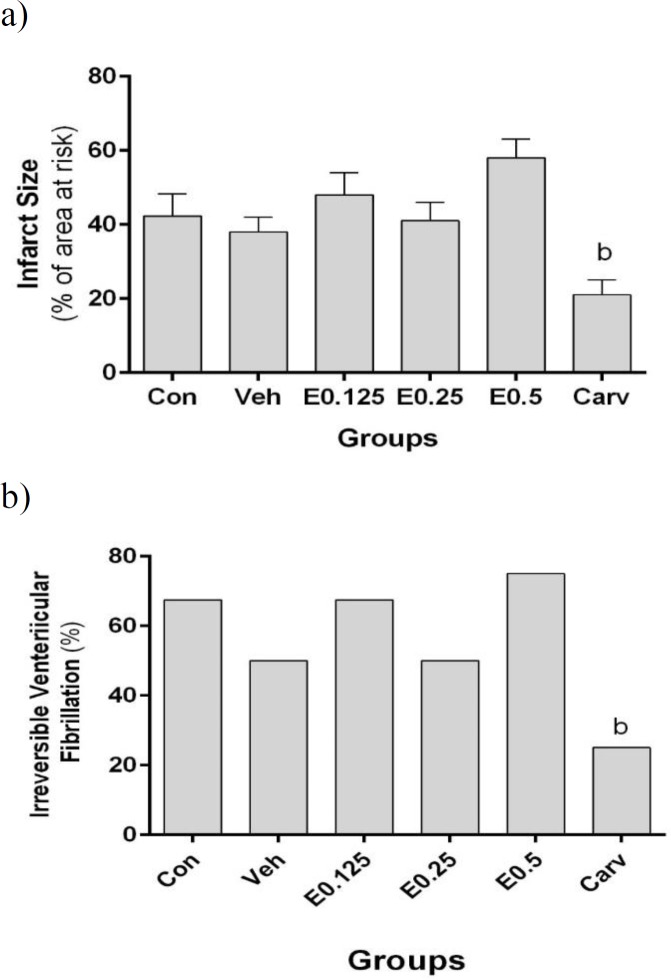
The effect of AEO on the incidence percentage of irreversible fibrillation (iVF) and the mean of percent change in infarct size following ischemic-reperfusion injury in isolated rat hearts. (a) the incidence percentage of iVF during reperfusion; (b) the mean of percent change in infarct size. Con: control group; E0.125, E0.250 and E0.5: groups perfused with AEO 0.125, 0.250 and 0.50 µL/g heart in 50 ml Krebs solution immediately before global ischemia, respectively. Carv: carvedilol group (10 µM). Data are shown as mean±SEM. n=8 in each group. ^b^ p<0.01 and ^c^ p<0.001 show significant differences as compared to control group

Figure 2a demonstrates that the incidence percentage of irreversible fibrillation did not significantly vary among E0.125, E0.25, E0.5, and control group. It was significantly reduced in carvedilol group. It was 67.5, 50, 67.5, 50, 75 and 25 % in Con, Veh, E0.125, E0.25, E0.50 and carvedilol groups, respectively.

In terms of the infarct size, no significant differences were observed between the control group and the AEO groups (Figure 2b). Infarct size was only significantly smaller in the carvedilol group compared to control group.

**Effect of AEO on biochemical parameters of coronary effluent**

Table. 1 shows the effect of perfusion of hearts with AEO on the biochemical parameters of coronary effluent following global ischemic-reperfusion injury. The activities of CK and LDH enzymes and the content of MDA in the coronary effluent significantly increased in E0.50 group in comparison to the control group. The levels of CK and MDA as well as LDH activities were not significantly different among E0.125, E0.25 and control groups. However, CK and LDH activities and MDA levelwere significantly reduced in carvedilol group.

Table 1 also shows that compared to the control group, the nitrite content of coronary effluent was insignificantly reduced in the E0.50 group and increased in carvedilol group. There was no significant difference among E0.125, E0.25 and control group. 

**Table 1:** The effect of AEO on the biochemical parameters of coronary effluent following ischemic-reperfusion injury

**Parameter**	**Groups**					
	**Con**	**Veh**	**E0.125**	**E0.25**	**E0.50**	**Cav**
**LDH **(U/ml)	9.7±1.4	9.1±1.2	9.2±1.4	9.9±1.1c	11.5±1.7^a^	7.1±0.7^b^
**CK **(U/ml)	5.4±1.1	4.9±0.8	5.6±1.6	6.1±0.8	7.4±0.7^a^	2.7±0.6^c^
**MDA **(mmol/mg protein)	2.6±0.1	2.5±0.2	2.4±0.1	2.8±0.2	3.4±0.4^a^	1.5±0. 5^c^
**Nitrite **(µm/l)	13.6±0.3	13.2±0.4	12.7±0.5	11.9±0.4	9.1±0.2^a^	17.4±0.7^b^

Data are shown as Mean±SEM. n=8 in each group.LDH: lactate dehydrogenase; CK: creatine kinase; MDA: malondialdehyde; Con: Control group; Veh: vehicle group; Cav: carvedilol group (10 µM); E0.125, E0.25 and E0.50 mean that the hearts were perfused with AEO 0.125, 0.25 and 0.50 µl/Lg, respectively.

a p<0.05,

b p<0.01 and

c p<0.001 show significant differences as compared to control group.

## Discussion

In our previous study, we showed that pretreatment of rats with asafetida (25, 50 and 100 mg/kg for 4 weeks, orally) had cardioprotective effects at lower doses and cardiotoxic effects at higher doses (Esmailidehaj et al., 2014 ▶). Also, we recently reported that AEO has potent vasodilatory effect on rat thoracic aorta (Esmaeili et al., 2017 ▶). In the current study, direct effect of AEOwas tested on -reperfusion injury in isolated rat hearts. AEO is one of the three main fractions of asafoetida oleo gum resin which mainly contains sulfur compounds (Amalraj and Gopi, 2016 ▶). The present data showed that AEO has cardiotoxic activity at higher concentrations (>0.5 µL/g heart), but it has no effect on the cardiac function at lower concentrations (0.125 and 0.25 µL/g heart). The higher concentrations (≥0.5 µL/g heart) worsened the myocardial dysfunction induced by ischemic-reperfusion injury. On the other hand, carvedilol (10 µM) as a positive control, showed cardioprotective effects. 

Although it is obtained from several types of Ferula, asafoetida oleo gum resin is mainly derived from the exudates of roots and rhizomes of *Ferula asafoetida *Linn. (Iranshahy and Iranshahi, 2011 ▶). As mentioned above, the main compounds of asafoetida essential oil are sulfur containing compounds (Kavoosi and Rowshan, 2013 ▶; El Deeb et al., 2012 ▶; Amalraj and Gopi, 2016 ▶). Sulfur compounds of asafoetida have valuable biological activities (Amalraj and Gopi, 2016 ▶). Recently, we reported that the main constituents of essential oil were di-(2-methyl-1,3-oxathiolanyl)methane (22.43%), trans-propenyl sec-butyl disulfide (14.59%), thiophene, 2-ethyltetrahydro- (10.61%), trans, trans-dibenzylideneacetone (10.07), cis-propnyl sec-butyl disulfide (8.78%), 2-methyl-2 methylthiopropionic acid (8.07%) and disulfide, methyl 1-(methylthio)propyl (5.54%) (Esmaeili et al., 2017 ▶). Kavoosi and co-workers reported variations in the composition of AEO among the species collected on 15th of June, 30th of June and 15th of July in 2011 (Kavoosi and Rowshan, 2013 ▶). The results of this study were partly consistent with the compositions of essential oil obtained from samples collected on 15th and 30th of June in their study but inconsistent with other reports (Dehpour et al., 2009 ▶; Bahrami et al., 2013 ▶; Hadavand Mirzaei and Hasanloo, 2014 ▶; Kavoosi and Purfard, 2013 ▶). These differences can be attributed to the conditions of culture, nutritional, climate, pollution and GC-MS temperature, the time of collection, type of asafoetida, and parts of the plant used (Dehpour et al., 2009 ▶; Hadavand Mirzaei and Hasanloo, 2014 ▶; Kavoosi and Purfard, 2013 ▶; Bamoniri and Mazoochi, 2009 ▶; Moghaddam et al., 2003 ▶). 

Previous studies have shown that asafoetida has antioxidant effect (Kavoosi et al., 2013 ▶; Mallikarjuna et al., 2003 ▶; Safari et al., 2016 ▶). Also, we observed that AEO has antioxidant effect against DDPH free radicals *in vitro* (data not shown). For this reason, it was hypothesized that perfusion of isolated rat hearts, prior to ischemic-reperfusion injury, with solution containing AEO may have cardioprotective effects. 

It has been documented that the increased production of free radicals especially at the beginning of reperfusion accounts for tissue damage following ischemia-reperfusion injury (Anaya-Prado et al., 2002 ▶; Moukarbel et al., 2004 ▶). Antioxidant therapy has been relatively successful in attenuation of this type of tissue injury (Anaya-Prado et al., 2002 ▶; Moens et al., 2005 ▶). Then, due to antioxidant activity of AEO, it was speculated that it might protect the cardiomyocytes against myocardial ischemia-reperfusion injury in isolated hearts through its free-radicals scavenging property. Our data showed that AEO 0.5 µL/g heart worsened the severity of -reperfusion injury. AEO had no effect on the severity of -reperfusion injury at the concentrations of 0.125 and 0.25 µL/g heart. These detrimental effects of AEO were indicated by increased LVEDP and decreased LVDP, ±dp/dt and coronary flow. When the hearts were perfused with AEO 1 µL/g heart, bradycardia and cardiac arrest immediately occurred. On the other hand, as the content of AEO in solution decreased to 0.5 µL/g heart, its detrimental effects were significantly reduced and at lower concentrations AEO did not have cardiotoxic effects. Cardiac functional parameters did not show any significant difference between control and E0.125 group. It appears that the effect of cardiotoxic compounds of AEO was more potent than that of cardioprotective and antioxidant compounds. It can be postulated that if the content of essential oil in the perfusion solution is lowered, its antioxidant activity could neutralize the effects of its cardiotoxic compounds. On the other hand, we assume that at lower concentrations (less than 0.125 µL/g heart), the essential oil might protect the heart against -reperfusion injury. These results conform the results of our pervious study (Esmailidehaj et al., 2014 ▶).

In 2004, Fatehi and co-workers reported that intravenous administration of asafoetida (0.3-2.2 mg/body weight) had hypotensive effect in anesthetized rats (Fatehi et al., 2004 ▶). They suggested that this effect might be mediated through the vasodilatory effect of asafoetida (Fatehi et al., 2004 ▶). Other studies using isolated rings of ileum and trachea of guinea pig have shown that AEO could relax the precontracted smooth muscles (Fatehi et al., 2004 ▶; Bayrami et al., 2013 ▶; Kiyanmehr et al., 2016 ▶). Recently, we also reported the vasodilatory effect of AEO on isolated rat thoracic aorta rings (Esmaeili et al., 2017 ▶). In the present study, perfusion of the hearts with AEO not only did not improve the coronary flow, but also it significantly reduced coronary flow at the concentration of 0.5 µL/g heart. It appears that asafoetida at this concentration destroyed the endothelium of coronary arteries. It is likely that asafoetida at very low concentrations (<0.125 µL/g heart) might lead to vasodilation and at high concentrations, it might lead to vasoconstriction through endothelium disruption. To confirm these results, we used carvedilol as a substance that can significantly increase coronary flow. Carvedilol is a selective alpha-adrenergic blocker and a nonselective beta-blocker that reduces cardiac morbidity and mortality in acute myocardial infarction and heart failure (DiNicolantonio et al., 2013 ▶; Li et al., 2017 ▶). It has also antioxidant, anti-inflammatory and immunomodulatory and anti-infarct effects (Brunvand et al., 1996 ▶). It has been reported that carvedilol but not propranolol, has potent antioxidant effects and significantly attenuates the intracellular concentration of calcium which completely inhibits hypercontracture in rat myocardium (Nakamura et al., 2009 ▶

The size of infarct did not differ significantly among the control and AEO-treated groups. These data are in accordance with the results of our previous study done in anesthetized rats (Esmailidehaj et al., 2014 ▶). To the best of our knowledge, there is no other study about asafoetida and the heart. The infarct size data are consistent with that of biochemical parameters, including the activities of LDH and CK enzymes and MDA and NO levels in the coronary effluent (Table 1). Also, there was no significant difference in the incidence of irreversible ventricular fibrillation between groups received AEO and control group. However, it was significantly decreased in carvedilol groups (Table. 2). 

Like other studies (Kavoosi and Rowshan, 2013 ▶; Kavoosi et al., 2013 ▶), AEO had antioxidant property in the present study, but the contents of MDA and nitrite in the coronary effluent did not show any significant difference among control, E0.125 and 0.25 groups. MDA and NO levels were significantly increased in E0.50 group but decreased in carvedilol group. In 2015, Korashy et al. reported that asafoetida inhibited the mRNA expression of CYP2C11 in the liver of rats treated with increasing doses of Ferula asafoetida. They proposed that asafoetida might lead to undesirable pharmacological effects of drugs which use CYP2C11 as a substrate (Korashy et al., 2015 ▶). Our results are inconsistent with other reports that might be due to differences in the methods and models used. Recently, Safari and colleagues reported that feeding of carp with asafoetida complementary diet, leads to increases in the expression of antioxidant enzymes GSR, GPx and GSTA (Safari et al., 2016 ▶). Keshri and co-workers reported that antifertility effect of asafoetida in rats is mediated through inhibition of several enzymes in the mitochondrial Krebs cycle (Keshri et al., 2004 ▶). According to these data, we suggest that asafoetida at high concentrations (> 0.5 µL/ g heart) might worsen the cardiac dysfunction by interrupting oxidative metabolism pathways in cardiomyocytes. It must be clarified in future studies. 

In summary, perfusion of isolated rat hearts with AEO (at concentrations ≥0.5 µL/ g heart) worsened the cardiac dysfunction following prolonged -reperfusion injury. Since lower concentrations of AEO had no cardiotoxic effects, it is recommended that the effect of lower concentrations of AEO should be assessed on the complications of acute myocardial infarction. 
